# Strengthening Children’s Advertising Defenses: The Effects of Forewarning of Commercial and Manipulative Intent

**DOI:** 10.3389/fpsyg.2016.01186

**Published:** 2016-08-08

**Authors:** Esther Rozendaal, Laura Buijs, Eva A. van Reijmersdal

**Affiliations:** ^1^Behavioural Science Institute, Radboud UniversityNijmegen, Netherlands; ^2^Amsterdam School of Communication Research, University of AmsterdamAmsterdam, Netherlands

**Keywords:** advertising, children, advertising literacy, forewarning, prompting, persuasion

## Abstract

This study investigated whether a forewarning of advertising’s intent can increase children’s (*N* = 159, 8–10 years old) defenses against television commercials to lower their desire for advertised products. Two different forewarnings were tested, one for advertising’s *commercial intent* or warning for the promotional nature, and one for advertising’s *manipulative intent* or warning for the deceptive nature. Results showed that only the warning of manipulative intent prior to advertising exposure was successful in increasing children’s advertising defenses. This forewarning activated children’s attitudinal advertising literacy (i.e., skepticism toward the commercial), which in turn led to lower advertised product desire. The forewarning of commercial intent was not effective in strengthening children’s advertising defenses. These findings have important implications for interventions that aim to lower children’s desire for (unhealthy) advertised products by activating their advertising literacy.

## Introduction

Today’s children face a media environment filled with advertising (Calvert, 2008; Buijzen et al., 2010). The commercialization of children’s media has raised serious concerns about its undesirable consequences for their wellbeing (i.e., materialism, unhealthy eating habits; Harris et al., 2011; Montgomery and Chester, 2011; Montgomery et al., 2011). Additionally, issues of fairness have been raised because children’s understanding of advertising and their critical attitude toward it, also referred to as *conceptual and attitudinal advertising literacy*, are still developing (John, 1999; Wright et al., 2005). Moreover, even when children have acquired the necessary advertising literacy, the affect-based nature of contemporary advertising, in combination with children’s immature cognitive abilities, prevent them from autonomously activating their advertising literacy and using it as a critical defense against the impact of advertising (Rozendaal et al., 2011). To help children deal with advertising, there is an increasing call for intervention techniques that can help them activate their advertising literacy and use it as a defense mechanism.

One intervention technique that has been proposed for activating advertising literacy is forewarning of advertising’s intent (Boerman et al., 2012; Fransen and Fennis, 2014). Previous research on forewarnings has shown that when the intent of an advertising message is revealed (i.e., through warnings prior to exposure), people’s cognitive and affective defense mechanisms are activated, resulting in lowered persuasion such as reduced favorable thoughts, ad attitudes, or brand attitudes (Petty and Cacioppo, 1977, 1979; Jacks and Devine, 2000; Wei et al., 2008; Lee, 2010; Boerman et al., 2012). However, these studies were all based on adult samples. Children and adults process commercial content in different ways, thus the effect of forewarnings on children could differ from the effect on adults.

We focused on 8- to 10-year old children, as an extensive and long established body of research in both Europe and the United States has shown that most children in this age group have acquired a basic level of advertising literacy with regard to television commercials. Around the age of eight, the majority of children are able to recognize the difference between television advertising and programs and they demonstrate an increasing understanding of the intent of advertising (for reviews, see John, 1999; Kunkel et al., 2004).

The aim of this study is to investigate whether forewarning of advertising’s intent is an effective technique to strengthen children’s advertising defenses. Additionally, the study aims to explore *how* forewarnings can reduce children’s desire for advertised products by looking at activated conceptual and attitudinal advertising literacy as potential mediators. By testing the underlying mechanisms that might explain the effects of forewarnings, theoretical insights into the impact of forewarnings on children’s advertising processing are provided.

Forewarning is an intervention technique that can increase people’s advertising defenses by warning them about the content and/or intent of a certain message (Chen et al., 1992). Overall, two types of forewarnings of advertising’s intent can be distinguished in the literature: forewarnings of advertising’s *commercial intent* (e.g., Boerman et al., 2012) and forewarnings of advertising’s *manipulative intent* (e.g., Sagarin et al., 2002).

A warning about *commercial intent* is a type of forewarning that informs people of the selling and persuasive intent of an advertising message. A warning of advertising’s commercial intent can increase people’s ability to resist the advertisement by informing them about the nature of the message, which enables them to arm themselves by activating their advertising literacy and using that literacy to prepare certain defense strategies (e.g., counterarguments), prior to exposure to the advertisement. Additionally, warning people that a message intends to persuade them can increase their motivation to resist by inducing feelings of psychological reactance (Brehm, 1966; Hass and Grady, 1975; Fukada, 1986; Fransen et al., 2015). Warnings about the commercial intent of an advertisement can cause people to feel restricted in their freedom to feel and think what they want. As a consequence, they are motivated to actively restore this freedom (Fransen and Fennis, 2014; Fransen et al., 2015). Previous research on adults indeed demonstrated that when people are warned about the persuasive and selling intent of a message, they are better able and more motivated to defend against the persuasive appeal of the message (Chen et al., 1992; Jacks and Devine, 2000; Lee, 2010; Boerman et al., 2012; Dekker and Van Reijmersdal, 2013).

The effects of a forewarning about commercial intent prior to exposure to a persuasive message can be best explained by cognitive processing mechanisms. Specifically, a forewarning about advertising’s commercial intent can serve as a cue that activates people’s conceptual advertising literacy by raising their awareness of the advertisement’s selling and persuasive intent (Boerman et al., 2012). This increased awareness of the commercial nature of an advertising message will increase people’s ability and motivation to process the message on a more cognitive level and to come up with counterarguments (Papageorgis, 1968; Petty and Cacioppo, 1977, 1979). These counterarguments will lead to less favorable attitudes toward the message (Jacks and Devine, 2000), and lowered product desires (Lee, 2010; Boerman et al., 2012). Therefore, it is expected that a forewarning of advertising’s commercial intent will reduce advertised product desire by activating a cognition-based defense mechanism called conceptual advertising literacy (i.e., increased awareness of the selling and persuasive intent of an advertisement).

A warning about *manipulative intent* is a type of forewarning that informs people of the fact that advertising can be deceptive. A forewarning of manipulative intent differs from a forewarning of commercial intent, in that the warning of commercial intent just informs about the fact that the messages is intended to sell and persuade, which is not necessarily deceptive. A forewarning of manipulative intent also warns people that a message intends to influence them, but adds an evaluative component in stating that they might be persuaded in a deceptive manner. The awareness that ads can be deceptive or manipulative can be an important motivator of advertisement resistance (Sagarin et al., 2002). Although research on forewarnings about manipulative intent is scarce, several studies have shown that the effectiveness of persuasive messages decreases if people perceive these messages as manipulative (MacKenzie and Lutz, 1989; Campbell, 1995; Ellen et al., 2000; Wentzel et al., 2010). Therefore, it is expected that a warning about the manipulative intent of advertising will be effective in stimulating resistance toward a persuasive message as it may activate perception of manipulative intent.

Contrary to the cognitive mechanisms that explain the effects of forewarnings about commercial intentions, the effects of forewarnings about manipulative intentions are expected to be caused by an affective mechanism. A warning about manipulative intent motivates resistance by arousing an unpleasant feeling within the viewer that they are being unfairly manipulated (Sagarin et al., 2002). As a consequence, people will activate their attitudinal advertising literacy by generating negative and skeptical feelings because they don’t like the idea of being misled. Attitudinal advertising literacy comprises of two elements: disliking, which is defined as a negative attitude toward advertising, and skepticism, which is defined as the tendency to disbelief advertising (Rozendaal et al., 2011). Based on advertising effect studies (Mitchell and Olson, 1981; Batra and Ray, 1986; Derbaix and Bree, 1997; Buijzen, 2007), activation of attitudinal advertising literacy is expected to lower advertised product preferences and purchase intentions, and thus result in decreased persuasion. Thus, a forewarning of advertising’s manipulative intent is expected to reduce children’s advertised product desire by activating the affective defense mechanism known as attitudinal advertising literacy (i.e., affective defense mechanism).

Despite the fact that the forewarning literature is primarily focused on adults, we expect that children have an even greater need of a forewarning to help them defend against advertising. Theories on children’s advertising processing (Moses and Baldwin, 2005; Nairn and Fine, 2008; Buijzen et al., 2010; Rozendaal et al., 2011), in which a developmental perspective on adult persuasion models is adopted, suggest that children primarily process advertising under conditions of low elaboration due to the affect-based nature of advertising. As a consequence, when confronted with advertisements, they are unlikely to autonomously activate and use their advertising literacy as a defense mechanism.

Furthermore, these theories suggest that children’s ability to use their advertising literacy as a defense will be further limited by their immature cognitive abilities. Children under the age of 12 have been labeled as “cued processors,” (Roedder, 1981; Brucks et al., 1988) which means that, due to their limited working memory capacity, these children need to be cued in order to retrieve and activate their stored knowledge. Therefore, in the context of this study, it is expected that a forewarning of advertising’s intent can function as a cue for children to activate their advertising literacy and to use it as a defense mechanism so as to be less susceptible to advertising effects.

To test the effectiveness of the two types of forewarnings (i.e., forewarning of commercial and manipulative intent) on children’s advertised product desire, we formulated the following hypothesis:

H1: Compared to no forewarning, (a) a forewarning of advertising’s commercial intent and (b) a forewarning of advertising’s manipulative intent lead to less advertised product desire.

Because we expect that the effect of a forewarning of commercial intent on children’s advertised product desire (as hypothesized in H1a) is mediated by their conceptual advertising literacy, we also formulate the following hypothesis:

H2: Compared to no forewarning or a forewarning of manipulative intent, a forewarning of commercial intent leads to higher awareness of (a) the selling intent and (b) the persuasive intent of the advertisement, which in turn leads to less advertised product desire.

Additionally, we expect that the effect of a forewarning of manipulative intent on children’s advertised product desire (as hypothesized in H1b) is mediated by their attitudinal advertising literacy. Therefore, we hypothesize the following:

H3: Compared to no forewarning or a forewarning of commercial intent, a forewarning of manipulative intent leads to higher (a) skepticism toward and (b) dislike of the advertisement, which in turn leads to less advertised product desire.

The current study also aims to investigate whether forewarning alone is enough to stimulate children’s advertising defenses or whether an additional prompt during commercial exposure that reminds them of the forewarning is needed. Due to the appealing emotional features in today’s advertising (Buijzen and Valkenburg, 2002), children are often so distracted and ‘soaked-up’ in advertisements that they are unlikely to naturally activate and use their advertising literacy (Livingstone and Helsper, 2006; Nairn and Fine, 2008; Harris et al., 2009; Buijzen et al., 2010; Rozendaal et al., 2011). Additionally, because children still have difficulty controlling their impulsive responses (i.e., lack of inhibitory control) and lack the ability to shift their attention away from appealing advertisements (i.e., lack of cognitive flexibility; Zelazo et al., 2003; Diamond, 2012), they are more likely to immediately respond to the salient features of an advertisement and less likely to use their advertising literacy as a defense mechanism (Rozendaal et al., 2011). Thus, even if they have been forewarned about advertising intent, whether either commercial or manipulative, children may forget about these warnings when exposed to highly appealing advertisements. Therefore, in addition to the forewarning, children may need an extra prompt that reminds them of the forewarning during advertising exposure to increase and activate their advertising literacy. A prompt is a recognizable sign that is clearly related to the forewarning, which is shown during advertising exposure. It is expected that a prompt will remind children of the forewarning, which in turn helps them to activate their advertising literacy and use it to critically process the advertisement. We therefore hypothesize the following moderated mediation effects:

H4: The mediated effect of a forewarning of commercial intent on advertised product desire via awareness of (a) the selling intent and (b) the persuasive intent of the advertisement (as hypothesized in H2) is moderated by an additional prompt, such that the mediated effect is stronger with a prompt than without a prompt.
H5: The mediated effect of a forewarning of manipulative intent on advertised product desire via (a) skepticism toward and (b) dislike of the advertisement (as hypothesized in H3) is moderated by an additional prompt, such that the mediated effect is stronger with a prompt than without a prompt.

## Materials and Methods

### Participants and Design

This study employed a 2 (forewarning: commercial intent vs. manipulative intent) × 2 (prompt: yes vs. no), factorial between-subjects design including a stand-alone control group (i.e., a baseline group that was not exposed to a forewarning or a prompt, but was exposed to the same program and commercials as the other conditions). A total of 159 children of 8- to 10-years-old (*M* = 8.73, *SD* = 0.72, 45% boys) participated in the experiment and were randomly assigned to one of the five conditions. The children were recruited from the third grade (*n* = 77) and fourth grade (*n* = 82) classes of two elementary schools located at a medium-sized city in the east of the Netherlands, and at a small city in the west of the Netherlands. The children were told that the aim of the study was to gather insights into their television preferences. The study was carried out in accordance with the recommendations of the Ethics Committee of the Amsterdam School of Communication Research, the University of Amsterdam, with written informed consent from all the children, their parents, and the schools. All subjects gave written informed consent in accordance with the Declaration of Helsinki.

### Materials

In order to test the effects of the forewarnings and the extra prompt during advertising exposure, we compiled a short video that included a fragment of *Spangas* (a popular children’s television series) followed by a commercial break; the commercial break consisted of a commercial bumper and three commercials: one for the *ABN AMRO* bank, one for the chocolate spread brand *Nutella*, and one for *Glorix* toilet cleaner. The *Nutella* commercial was the stimulus commercial of our study, the other two commercials were fillers. Thus, all variables (see Measures) were measured for the *Nutella* commercial only. The *Nutella* commercial was selected as the stimulus commercial for three reasons: (1) the advertised product category (i.e., chocolate spread) was appealing for most children, (2) the commercial was gender neutral, and (3) it targeted children in our age group. We focus on television commercials because children still spend a vast amount of leisure time watching television (Ofcom, 2014; Opree et al., 2014).

The program *Spangas* shows how a group of secondary school students deal with school, their friends, and classmates, and was selected because it targets children in the same age group as our sample, and it is appealing for both boys and girls (Rozendaal et al., 2012). To render the viewing situation as naturally as possible, we included a commercial bumper that is typically used on the television channel broadcasting *Spangas*. This bumper showed a short animation of a character named Olec and the name of the television channel. No explicit announcement referring to advertising or the start of a commercial break was made. All children were exposed to the same video compilation. However, the children in the experimental group were also exposed to one of the two advertising forewarnings. Additionally, half of these children were presented with an additional prompt while viewing the commercials.

#### Forewarnings about Advertising’s Intent

The two different forewarnings were presented during the commercial bumper prior to the commercials. The commercial intent forewarning said, “Now it’s time for the commercials, but pay attention: commercials want you to like and buy their products.” The manipulative intent forewarning said, “Now it’s time for the commercials, but pay attention: commercials are not always fair, sometimes they tell things that are untrue.” This was done both in text and in audio via voice over to help children understand the meaning of the forewarning (An and Stern, 2011). The character *Olec* was used as the forewarning spokesperson (i.e., he was the one who warned children about the upcoming commercials). The Olec character, a gray with black spots child-friendly monster, has been developed by the channel broadcasting *Spangas* and is specifically used in commercial bumpers for the purpose of program-commercial separation.

#### Prompt

The intent of the additional prompt was to remind children of the advertising forewarning presented by Olec, and consisted of a small visual of Olec with a red flag in his hand that appeared during the advertisement in order to attract the children’s attention. In compliance with recommendations of the English Federal Office of Communications (Ofcom) about the appearance of advertising-related logos on television (i.e., product placement transparency), the prompt was presented for 3 s in the upper right corner of the screen during all three commercials (Ofcom, 2010).

### Procedure

Each child was placed in front of a personal computer within a room with more computers and participants. All children were instructed to put their headsets on and watch the video compilation. Afterward, they filled out an online questionnaire. Survey questions included demographic information, frequency of watching television, attitude toward the channel that broadcasts *Spangas*, attitude toward the television program *Spangas.* Advertised product desire, conceptual and attitudinal advertising literacy were also assessed, and children indicated their attitude toward Olec, familiarity with the brand *Nutella*, and how often they ate *Nutella*. Finally, children were asked what they thought the research was about. The children were also told that they were allowed to halt participation at any time.

### Measures

#### Advertised Product Desire

To assess children’s *advertised product desire* we asked two questions: “Do you want to have *Nutella* chocolate spread?”, and “Will you ask your parents to buy *Nutella* chocolate spread for you?” The answers were rated on a four-point scale: 1 (*no, certainly not*), 2 (*no, I don’t think so*), 3 (*yes, I think so*), and 4 (*yes, certainly*) (Rozendaal et al., 2013) (Cronbach’s α = 0.77; [Pearson’s] *r* = 0.63; *M* = 2.79, *SD* = 0.92). With advertised product desire we grasp both children’s longing for the advertised product and their intended purchase request, which has proven to be good indicators for family purchase decisions (Swinyard and Sim, 1987; Valkenburg, 2004).

The correlation coefficient (*r* = 0.63) shows a moderate relationship between the two items. This relatively low score could be a result of the fact that it is more difficult to measure the responses of children than of adults. Many other studies that have focused on children as participants have found comparable inter-item correlation scores (for example, see van Reijmersdal et al., 2012; Rozendaal et al., 2013; Opree et al., 2014) indicating that moderate inter-item correlation scores are common when doing research with children.

#### Activated Conceptual Advertising Literacy

Based on the work of Rozendaal et al. (2016), we measured two components of conceptual advertising literacy. Specifically, we aimed to assess children’s knowledge of advertising, in particular whether they know why commercials (in this case *Nutella*) are being broadcast. First, *awareness of the advertisement’s selling intent* was measured with a single-item question: “Is this commercial on TV to make you buy *Nutella* chocolate spread?” The answers were rated on a four-point scale: 1 (*no, certainly not*), 2 (*no, I don’t think so*), 3 (*yes, I think so*), and 4 (*yes, certainly*) (*M* = 3.39, *SD* = 0.86). Second, *awareness of the advertisement’s persuasive intent* was measured with a single-item question: “Is this commercial on TV to make you like *Nutella* chocolate spread?” The same four-point scale that was used to rate this question (*M* = 2.92, *SD* = 0.92).

#### Activated Attitudinal Advertising Literacy

Based on the work of Rozendaal et al. (2016), two components of attitudinal advertising literacy were measured. First, *skepticism toward the advertisement* was measured with five questions: “Do you think the commercial for *Nutella* chocolate spread is fair?” (reverse); “Do you think the commercial for *Nutella* chocolate spread tells things that are untrue?”; “Do you think the commercial for *Nutella* chocolate spread tells the truth?” (reverse); “Do you think the commercial for *Nutella* chocolate spread is lying?”; and “Do you think you can believe the commercial for *Nutella* chocolate spread?” (reverse). The answers were rated on a four-point scale: 1 (*no, certainly not*), 2 (*no, I don’t think so*), 3 (*yes, I think so*), and 4 (*yes, certainly*) (Cronbach’s α = 0.82, *M* = 2.45, *SD* = 0.70).

Second, *dislike of the advertisement* was measured with six questions: “Do you think the commercial for *Nutella* chocolate spread is nice?” (reverse), “Do you think the commercial for *Nutella* chocolate spread is funny?” (reverse); “Do you think the commercial for *Nutella* chocolate spread is boring?”; “Do you think the commercial for *Nutella* chocolate spread is beautiful?” (reverse); “Do you think the commercial for *Nutella* chocolate spread is stupid?”; and “Do you think the commercial for *Nutella* chocolate spread is ugly?” The answers were given on the following scale: 1 (*no, not at all*), 2 (*no, not really*), 3 (*yes, a little bit*), and 4 (*yes, very much*) (Cronbach’s α = 0.84, *M* = 2.35, *SD* = 0.71).

It might be important to note that, because the variations in our independent variable (i.e., forewarning type) are defined in terms of intrinsic features (both forewarnings were demonstrably different; whether the forewarning referred to the promotional or deceptive nature of advertising is not a matter of participants’ perceptions), children’s skepticism toward and dislike of the advertisement were understood and analyzed as mediating variables and not as manipulation checks (see O’Keefe, 2003).

#### Background Characteristics

Various background characteristics were measured to check that intervention effects were not caused by other differences between the experimental groups. Based on the affect transfer principle (Shimp, 1981), we expected that children’s attitude toward the channel broadcaster, their attitude toward the television program *Spangas*, and their attitude toward the television character Olec would impact their responses toward the forewarning and the commercials. Therefore, we asked participants how much they liked the channel broadcaster (*M* = 3.00, *SD* = 0.76), *Spangas* (*M* = 3.23, *SD* = 0.75), and Olec (*M* = 3.14, *SD* = 0.81). We again used a four-point scale: 1 (*no, not at all*), 2 (*no, not really*), 3 (*yes, a little bit*), and 4 (*yes, very much*). Furthermore, based on the persuasion effect literature (Chattopadhyay and Basu, 1990; Campbell, 1995), we expected that initial familiarity with a brand would affect persuasion. Therefore, we asked participants how familiar they were with the brand *Nutella* (88% said yes) and with *Nutella* commercials (80% said yes). We also asked how often they ate *Nutella* chocolate spread (*M* = 2.06, *SD* = 0.89), using the following scale: 1 (*never*), 2 (*sometimes*), 3 (*often*), and 4 (*very often*). In addition, because the advertising literacy literature (Rozendaal et al., 2009, 2010) predicts that the frequency of television watching is related to children’s advertising literacy levels, we asked how often they watched television (*M* = 2.99, *SD* = 0.76), and how often they watched the channel that hosted *Spangas* (*M* = 2.50, *SD* = 1.06); questions were rated on a four-point scale that ranged from 1 (*never*) to 4 (*very often*). Finally, sex, age, and grade were obtained as well.

## Results

### Randomization

Before testing our hypotheses, we conducted randomization checks with a one-factor analysis of variance (ANOVA) for all background variables. The analysis showed no differences in the four experimental groups and the control group with respect to the dependent variables. To further examine which variables should be used as covariates, we conducted correlational analyses. Because these analyses showed that sex, grade, attitude toward the channel that broadcasts *Spangas*, frequency of watching television, and frequency of eating *Nutella* chocolate spread were related to the dependent variable, these variables were included as covariates in all the analyses. Pearson’s *r* correlations between the covariates and the dependent variables ranged between -0.22 and -0.19 and between 0.18 and 0.44. The covariate “attitude toward the channel that broadcasts *Spangas*” had 13 uncategorized responses because these children were not familiar with the channel. Therefore, these children will be excluded, and all further analyses will be conducted with a total sample of 146 (initial sample of 159 minus these 13 children).

### Testing of Hypotheses

To test H1, that stated that compared to no forewarning, (a) a forewarning of advertising’s commercial intent and (b) a forewarning of advertising’s manipulative intent would lead to less advertised product desire, we conducted a univariate analysis of covariance. Advertised product desire was entered as the dependent variable and all five conditions as the independent variable. The analyses showed no significant effect of the conditions, *F*(4,136) = 1.44, *p* = 0.23, η_p_^2^ = 0.04. Therefore, H1 on the effect of forewarning types on advertised product desire must be rejected.

To test H2 and H3 that stated that the effects of both forewarning types on children’s advertised product desire would be mediated by children’s activated advertising literacy; we first conducted a multivariate analysis of covariance. The four mediators (e.g., awareness of advertisement’s selling intent, awareness of advertisement’s persuasive intent, skepticism toward the advertisement and disliking of the advertisement) were entered as the dependent variables and all five conditions as the independent variable. This analysis showed a significant multivariate effect of the groups, Wilk’s λ = 0.71, *F*(16,406) = 3.08, *p* < 0.001, η_p_^2^ = 0.08. The univariate analyses showed that the only significant effects of the groups was on skepticism toward advertising, *F*(4,136) = 8.36, *p* < 0.001, η_p_^2^ = 0.20, see **Table 1** *Post hoc* analyses showed that the groups that saw a forewarning for manipulative intent had significantly higher levels of skepticism toward the television commercial than the groups that received a forewarning of commercial intent (mean difference = 0.70, *SE* = 0.17, *p* = 0.001) or no forewarning (mean difference = 0.59, *SE* = 0.17, *p* = 0.006). Also, the group that received a forewarning of manipulative intent plus a prompt scored significant higher on skepticism than the groups that received a forewarning of commercial intent (mean difference = 0.79, *SE* = 0.17, *p <* 0.001), or no forewarning (mean difference = 0.68, *SE* = 0.17, *p* = 0.001). No differences were found between the groups that saw a forewarning of manipulative intent with and without the prompt (mean difference = 0.09, *SE* = 0.17, *p* = 1.00). Also no differences were found between the groups that saw a forewarning of commercial intent with and without the prompt (mean difference = 0.40, *SE* = 0.17, *p* = 0.20). Thus, for both forewarning types, the prompt had no effect.

**Table 1:** Means scores on advertised product desire and the mediators for the forewarning conditions and control group.

	Forewarning				
	No	Commercial intent	Commercial intent + prompt	Manipulative intent	Manipulative intent + prompt
Advertised product desire	3.07 (0.16)	2.76 (0.16)	2.89 (0.15)	2.60 (0.15)	2.68 (0.15)
Awareness of selling intent	3.27 (0.16)	3.24 (0.17)	3.60 (0.15)	3.66 (0.16)	3.10 (0.16)
Awareness of persuasive intent	2.67 (0.17)	3.25 (0.18)	2.98 (0.16)	3.07 (0.17)	2.69 (0.17)
Skepticism toward advertising	2.17^a^ (0.12)	2.06^a^ (0.12)	2.46^ab^ (0.11)	2.76^b^ (0.12)	2.85^b^ (0.12)
Disliking of advertising	2.17 (0.14)	2.17 (0.14)	2.44 (0.13)	2.56 (0.14)	2.40 (0.14)

*Estimated marginal means are portrayed with (standard deviations between parentheses). ^a^Means with different superscripts in the same row differ significantly from each other in Bonferonni tests at *p* < 0.05.*

This means that H2 that stated that forewarning of commercial intent (vs. forewarning of manipulative intent or no forewarning) would lead to higher awareness of selling or persuasive intent and consequently to less advertised product desire is rejected. Similarly, H3b on the effects of forewarning of manipulative (vs. forewarning of commercial intent or no forewarning) on advertised product desire through disliking of advertising is rejected, because disliking of advertising was not affected by the forewarnings.

Because the comparison of the five groups did show significant differences between the forewarning types with respect to skepticism, further analyses were conducted to test H3a. Indirect effects instead of mediated effects of forewarning of manipulative intent through skepticism are tested, because the previous analysis showed no direct effect of forewarning on advertised product desire, In order to test the hypothesized indirect effects, we used Hayes’ (2013) bootstrap approach (PROCESS, model 4, 99% bias corrected accelerated confidence intervals), with 10,000 bootstrap resamples. The confidence interval was adjusted to control for the multiple comparisons that were made to compare all three forewarning types. The same covariates as before were included. The mediator, dependent variable and covariates were standardized to facilitate the interpretation of the effects.

First, we compared the forewarning of manipulative intent to the control group. We conducted an indirect effects analysis with forewarning of manipulative intent (vs. the control group) as the independent variable, advertised product desire as the dependent variable, skepticism as the mediating variable, and a dummy variable for the forewarning of commercial intent as a covariate. The analysis showed a significant indirect effect of forewarning of manipulative intent (compared to no forewarning) on advertised product desire, through skepticism; see **Table 2** (total indirect effect). As shown in **Table 2**, the forewarning of manipulative intent resulted in significantly higher skepticism (*b* = 0.91, *SE* = 0.21) than no forewarning, which in turn resulted in significantly less advertised product desire (*b* = -0.26, *SE* = 0.07). Thus, as predicted compared to no forewarning, the forewarning of manipulative intent resulted in less advertised product desire through increased skepticism.

**Table 2:** Mediation effects of forewarning of commercial intent and of manipulative intent via skepticism on advertised product desire.

		Effect						
	Intercept^1^	x on m	m on y	Direct	Total	Total indirect	*BCA* 99% CI [lower; upper]	Effect size
Forewarning								
Manipulative intent (1) vs. No Forewarning (0)	0.18	0.91^∗∗^ (0.21)	-0.26^∗∗^ (0.08)	-0.23 (0.21)	-0.46^∗^ (0.20)	**-0.24** **(0.09)**	-0.53; -0.05	-0.27
Manipulative intent (1) vs. Commercial intent (0)	-0.03	0.76^∗∗^ (0.17)	…	-0.01 (0.17)	-0.20 (0.16)	**-0.20** **(0.08)**	-0.44; -0.05	-0.23
Commercial intent (1) vs. No Forewarning (0)	0.19	0.15 (0.21)	…	-0.22 (0.20)	-0.26 (0.20)	-0.04 (0.06)	-0.21; 0.09	-0.05

***N* = 146.* b-coefficients (with boot SE between parentheses) are presented. ^∗^*p* < 0.05, ^∗∗^*p* < 0.01, total indirect effects in bold are significant at *p* < 0.01. … Scores are the same as the scores above. Effect size, partially standardized indirect effect sizes for the indirect effect are reported. ^1^Intercept of the model for effects on advertised product desire. Mediator, dependent variable and covariates are standardized. Model statistics for effects on skepticism, *F*(7,138) = 4.99, *p* < 0.001, *R*^*2*^= 0.20. Model statistics for effects on advertised product desire, *F*(7,138) = 7.86, *p* < 0.001, *R*^*2*^ = 0.29.*

Second, we compared the forewarning of manipulative intent to the forewarning of commercial intent by using forewarning of manipulative intent (vs. the forewarning for commercial intent) as the independent variable and a dummy variable for the control group as a covariate. The analysis showed a significant indirect effect of forewarning of manipulative intent (compared to forewarning of commercial intent) on advertised product desire, through skepticism, see **Table 2** (total indirect effect). As shown in **Table 2**, the forewarning of manipulative intent resulted in significantly higher skepticism (*b* = 0.76, *SE* = 0.17) than the forewarning of commercial intent, which in turn resulted in significantly less advertised product desire (*b* = -0.26, *SE* = 0.07). Thus, as predicted compared to the forewarning of commercial intent, the forewarning of manipulative intent resulted in less advertised product desire through increased skepticism. These two analyses provide support for H3a.

An additional analysis was conducted to compare the forewarning of commercial intent with no forewarning (with the forewarning of commercial intent as the independent variable and the dummy for forewarning of manipulative intent as a covariate). This analysis showed no significant indirect effect, see **Table 2** This means that the indirect effect of forewarning of commercial intent on advertised product desire through skepticism did not differ from the indirect effect of no forewarning. **Table 3** shows the effects of the covariates on skepticism and advertised product desire.

**Table 3:** Effects of covariates on skepticism and advertised product desire in mediation analysis.

Dependent variable	Skepticism			Advertised product desire		
	*b* (*SE*)	*t*	*p*	*b (SE)*	*t*	*p*
Sex (*F* = 1)	-0.11 (0.07)	-1.40	0.16	0.06 (0.07)	0.79	0.43
Grade	0.13 (0.08)	1.77	0.08	-0.13 (0.07)	-1.91	0.06
Frequency TV watching	-0.14 (0.08)	-1.84	0.06	-0.02 (0.07)	-0.22	0.82
Channel attitude	-0.05 (0.08)	-0.66	0.51	0.14 (0.07)	1.86	0.07
Frequency of eating *Nutella*	-0.07 (0.07)	-0.99	0.32	0.41 (0.07)	5.94	<0.001

*Covariates and dependent variables are standardized. Output is based on mediation analysis using PROCESS macro.*

With respect to H4 and H5 on the moderating influence of an additional prompt the previous univariate and multivariate analyses showed no significant effects of the prompt on the dependent (e.g., advertised product desire) and mediating variables (e.g., awareness of advertisement’s selling intent, awareness of advertisement’s persuasive intent, skepticism toward the advertisement and disliking of the advertisement). Thus, no significant differences were found between the forewarning type conditions with and without the prompt. Therefore, H4 and H5 are rejected: the mediated effect of forewarning type on advertised product desire is not moderated by the prompt.

## Discussion

This study investigated whether forewarning of advertising’s intent can strengthen children’s advertising defenses. Two different forewarnings were tested, one for advertising’s commercial intent (warning for the promotional nature) and one for advertising’s manipulative intent (warning for the deceptive nature). Specifically, we examined whether these forewarnings can help children activate their conceptual (i.e., understanding of the advertisement’s selling and persuasive intent) and attitudinal advertising literacy (i.e., skepticism toward and dislike of the advertisement), and whether this in turn leads to reduced advertised product desire.

Findings showed that only the forewarning of manipulative intent was effective in stimulating children’s advertising defenses. As expected, compared to no forewarning, the forewarning of manipulative intent increased children’s skepticism toward the advertisement, which in turn led to less advertised product desire. The forewarning of manipulative intent did not increase children’s awareness of the selling and persuasive intent of the advertisement. This indicates that a forewarning of manipulative intent is effective in reducing children’s advertised product desire by activating their attitudinal advertising literacy. This finding is in accordance with previous forewarning studies (Jacks and Devine, 2000; Lee, 2010) that have shown that when warnings result in skepticism, more resistance to persuasion follows (Boerman et al., 2012, 2014). Additionally, and most importantly, it also relates to the way children process advertising. Due to their immature cognitive abilities and the affect-based nature of advertising, children usually process advertising under conditions of low cognitive elaboration (Buijzen et al., 2010). This implies that children’s responses to an advertising message will be primarily based on affect instead of ratio. Because warnings about manipulative intent function via low-effort affective mechanisms such as feelings of skepticism that require low cognitive elaboration, these might be easier pathways for children to defend against the persuasive appeal of advertising.

Moreover, it’s possible that children process advertising at an even lower elaborate level at home as compared to in the classroom as was the case in our study. In real-life situations, the warning of manipulative intent is therefore expected to be most effective in reducing children’s advertised product desire. This forewarning naturally activates attitudinal advertising literacy more than conceptual advertising literacy.

Another explanation for the previously discussed forewarning effect relates to negative priming. That is, the forewarning of manipulative intent, which is a negatively valenced message, could have primed negative feelings toward the advertisement. However, we only found an effect of the warning of manipulative intent on skepticism, not on disliking. If negative priming would have occurred, the warning should have resulted in greater dislike of the advertisement as well, which is not the case.

Interestingly, the findings showed that the additional prompt did not further increase the effectiveness of the forewarning of manipulative intent. This suggests that a forewarning of advertising’s manipulative intent alone was sufficient to activate children’s attitudinal advertising literacy and lower their advertised product desire. No additional prompt was needed to reinforce their advertising defenses, if their attitudinal advertising literacy was already activated. However, maybe other prompts than used in this study, may increase the effects of forewarnings. It remains unknown whether prompts in general have no additional effect, or whether it is only the type of prompt that we used. Maybe the red flag was not informative enough for the children to exert an effect.

For the forewarning of commercial intent, our study showed some unexpected findings. Contrary to our expectations, the forewarning of commercial intent did not increase children’s awareness of the advertisement’s selling and persuasive intent, nor did it affect their advertised product desire. We also explored whether an extra prompt during advertising exposure could increase the effectiveness of the forewarning of commercial intent. The findings revealed that this was not the case. One explanation for this finding is that warnings about commercial intent require cognitive capacity (Fransen and Fennis, 2014) because retrieving and activating stored advertising knowledge, while one is exposed to highly appealing advertisements, is a form of self-regulation that requires high effort. In line with existing insights on children’s advertising processing (Livingstone and Helsper, 2006; Buijzen et al., 2010; Rozendaal et al., 2012) and cognitive development (Brucks et al., 1988; Moses and Baldwin, 2005), our results suggest that, even when forewarned about advertising’s commercial intent, children are unable or unmotivated to allocate a high level of cognitive resources in order to adopt the cognitive defense strategies needed to withstand the effects of advertisements.

In sum, the findings show that only the forewarning about manipulative intent was effective in stimulating children’s advertising defenses through the activation of their attitudinal advertising literacy. Specifically, the warning of manipulative intent increased children’s skepticism toward the commercial, which in turn led to lower advertised product desire. No additional prompt during advertising exposure was needed to reinforce this process. In contrast to expectations, the forewarning of commercial intent was not effective in lowering children’s advertised product desire. In line with earlier research (Zuwerink and Devine, 1996; Derbaix and Bree, 1997; Buijzen, 2007; Rozendaal et al., 2011, 2012), this suggests that children are best protected against advertising effects when their affective defenses are stimulated by increasing their attitudinal advertising literacy.

### Limitations and Directions for Further Research

Our study has some limitations regarding the research materials used and the effects studied. With respect to the materials, there are three limitations. First, our study focused on only two types of forewarnings: forewarning of commercial intent and forewarning of manipulative intent. Although these are the types of forewarnings that are most often discussed in the advertising and persuasion literature, in practice, other types of forewarnings could be used as well (e.g., forewarning of informative intent). Future research could examine a broader range of forewarnings to unravel whether different types of forewarnings lead to different effects.

Second, the present study focused on television advertising. Owen et al. (2013) demonstrated that children understand the intentions of traditional advertising (e.g., television commercials) better than non-traditional advertising (e.g., movie and in-game brand placements, advergames, and program sponsorships). It is likely that children experience even lower motivation and ability to defend against these embedded advertising formats, as compared to traditional advertising. Therefore, further research might examine the effectiveness of interventions aimed at reducing children’s advertised product desire in response to non-traditional, more embedded, advertising formats (see, e.g., An and Stern, 2011).

Third, our study showed the effects of using different types of forewarnings and of using a prompt vs. not using forewarnings or prompts. Because we did not include a control group that was not exposed to advertising, it remains unclear what the effects are of forewarnings and prompts compared to not being exposed to advertising. Such a control group could serve as a baseline for advertising exposure effects and should be included in future research.

With respect to the effects that were studied, there are two limitations regarding the endurance and types of effects. First, we did not include a follow-up measurement in our design. Children’s advertised product desire was measured immediately after exposure to the forewarning and the advertisements. Therefore, it remains unclear whether children’s resistance as evoked by the forewarning lasted for a longer period of time. Future research is needed to test the duration of forewarning effects among children.

Second, future studies could assess actual consumption behavior by employing a real choice situation in which the children get to pick real products, instead of asking for their advertised product desire through self-report. This could also give insights into the effects of the commercial and forewarnings on other unhealthy products than the one advertised (cf. Halford et al., 2004, 2007). In this study, a self-report measure was used instead of actual choice behavior, because the participating schools did not allow unhealthy foods in the class room. Due to an increasing awareness of overweight and obesity in children, increasingly more schools are adopting food policies that restrict unhealthy food products in school. This poses a challenge for future research focusing on children’s actual unhealthy food choices or eating behavior, because it will become more difficult to find schools that are willing to participate.

### Implications

Even with these limitations, this study has several theoretical and practical implications. At a theoretical level, the study contributes to our understanding of the role of forewarning’s of advertising’s intent in children’s advertising processing. Thus far, no studies have examined the effects of forewarnings among children. Our results show that forewarnings can be effective in stimulating children to process the advertisement more critically, resulting in lower advertised product desire. This finding is in line with earlier research among adults (Jacks and Devine, 2000; Sagarin et al., 2002; Lee, 2010; Boerman et al., 2012, 2014; Dekker and Van Reijmersdal, 2013). However, our study showed that only a forewarning for advertising’s manipulative intent was effective. This is in contrast with literature on adults, that shows that forewarning’s of advertising’s commercial intent are successful in stimulating adults’ advertising defenses as well. Moreover, the underlying mechanisms for the effects of forewarnings differ for children and adults. Whereas forewarning’s of advertising’s intent have been proven to be successful among adults because it activates their conceptual advertising literacy (i.e., *cognitive* mechanism), this was not the case with children. Our study showed that for children, forewarnings were most effective in inducing skeptical attitudes toward the ad (i.e., *affective* mechanism), which in turn led to lower advertised product desire. This implies that for children, forewarnings of advertising’s intent primarily function through attitudinal mechanisms. This is in line with theories on children’s advertising processing that predict that because children’s information processing capabilities are still developing, they primarily process advertising based on low-effort, attitudinal mechanisms (see, e.g., Moses and Baldwin, 2005; Buijzen et al., 2010; Rozendaal et al., 2011). Thus, the reason why only the warning of manipulative intent was effective in reducing children’s advertised product desire is that they naturally activate affective defense mechanisms more than cognitive defense mechanisms.

This article also has practical implications for the development of methods that decrease persuasion in children. Many countries have invested in the development of advertising education programs aimed at empowering children to defend against advertising. In many of these programs, the focus is on increasing children’s knowledge of advertising. However, this study shows that an increased conceptual advertising literacy does not suffice to diminish children’s advertised product desire. Efforts should be made to enhance children’s critical attitudes. As this study demonstrated, children’s skepticism toward advertising is critical to increasing their resistance to advertising persuasion.

This study also has several implications for public policy. Our findings suggest that forewarnings about advertising’s manipulative intent help children become (1) more critical toward advertising, and (2) lower their advertised product desire. Where the aim of policy makers is to decrease children’s advertised product desire (e.g., when unhealthy products are concerned), a forewarning about advertising’s manipulative intent offers potential. However, due to the negative valence of this warning, self-regulatory bodies of advertisers may not be supportive toward the implementation of this type of warning. Where the aim of policymakers is only to increase children’s awareness of advertising’s promotional nature, none of the investigated forewarning types are useful. Further research should reveal if other types of forewarnings or other types of interventions could be helpful in this respect.

## Conclusion

This study demonstrates that forewarning young children about advertising’s manipulative intent is a promising method to induce more critical advertising processing. Furthermore, the study demonstrates that in order to make children more resistant toward advertising, affective defense mechanisms (defined as activated attitudinal advertising literacy) are of huge importance.

## Author Contributions

LB, ER, and EvR conceived of the study. LB and ER wrote the paper. LB collected the data. EvR analyzed the data and commented on the paper. LB, ER, and EvR read and approved the final paper.

## Conflict of Interest Statement

The authors declare that the research was conducted in the absence of any commercial or financial relationships that could be construed as a potential conflict of interest.
